# Cancer Response to Therapy-Induced Senescence: A Matter of Dose and Timing

**DOI:** 10.3390/cancers13030484

**Published:** 2021-01-27

**Authors:** Maria Patrizia Mongiardi, Manuela Pellegrini, Roberto Pallini, Andrea Levi, Maria Laura Falchetti

**Affiliations:** 1 CNR-Institute of Biochemistry and Cell Biology, Campus Adriano Buzzati Traverso, Via Ercole Ramarini 32, Monterotondo Scalo, 00015 Rome, Italy; mariapatrizia.mongiardi@cnr.it (M.P.M.); manuela.pellegrini@cnr.it (M.P.); andrea.levi@cnr.it (A.L.); 2 Institute of Neurosurgery, Università Cattolica del Sacro Cuore, Largo Agostino Gemelli 8, 00168 Rome, Italy; roberto.pallini@unicatt.it

**Keywords:** senescence, cancer therapy, Senescence-Associated Secretory Phenotype (SASP), cancer cell, tumor vasculature

## Abstract

**Simple Summary:**

Cellular senescence consists of a permanent block of cell proliferation in the presence of an active metabolism. It is a physiological process occurring when cells exhaust their proliferative potential, as signaled by critical telomere erosion. Additionally, cell senescence might be triggered as a response to different stresses: DNA damage, oxidative stress and oncogenic activation. Whatever the senescence-inducing stress is, a peculiarity of senescent cells is the production of an altered secretome, the Senescence-Associated Secretory Phenotype (SASP), which profoundly affects the cellular microenvironment. Cancer therapy, either ionizing radiations or chemotherapy, induces cellular senescence, the so-called therapy-induced senescence (TIS). The issue of whether TIS is a pro- or anti-tumorigenic process is a current and open question.

**Abstract:**

Cellular senescence participates to fundamental processes like tissue remodeling in embryo development, wound healing and inhibition of preneoplastic cell growth. Most senescent cells display common hallmarks, among which the most characteristic is a permanent (or long lasting) arrest of cell division. However, upon senescence, different cell types acquire distinct phenotypes, which also depend on the specific inducing stimuli. Senescent cells are metabolically active and secrete a collection of growth factors, cytokines, proteases, and matrix-remodeling proteins collectively defined as senescence-associated secretory phenotype, SASP. Through SASP, senescent cells modify their microenvironment and engage in a dynamic dialog with neighbor cells. Senescence of neoplastic cells, at least temporarily, reduces tumor expansion, but SASP of senescent cancer cells as well as SASP of senescent stromal cells in the tumor microenvironment may promote the growth of more aggressive cancer subclones. Here, we will review recent data on the mechanisms and the consequences of cancer-therapy induced senescence, enlightening the potentiality and the risk of senescence inducing treatments.

## 1. Introduction

Cellular senescence is a complex phenomenon that occurs when cells exhaust their replication potential and in response to a variety of cellular stresses that reduce cell fitness [1,2]. It may be considered as an extreme form of cell differentiation, insofar as it requires substantial epigenetic modifications and chromatin remodeling, and affects gene expression profile in a partially stereotyped way [3,4].

Senescence is generally characterized by high expression of the cyclin-dependent kinase inhibitors p16INK4a and p21, lack of expression of the cell-cycle associated Ki67 protein, reduction of Lamin B1, and elevated level of trimethylated histone 3 lysine 9 (H3K9me3) often organized in foci, the senescence-associated heterochromatin foci, SAHF. Senescent cells show a flattened morphology with an enlarged lysosomal compartment accompanied with the expression of senescence-associated β-galactosidase (SA-β-gal) expression. Of note, not every senescent cell necessarily expresses all these markers, and many of them may be present in non-senescent cells. For instance, reduced Ki67 and elevated p16INK4a and p21 protein expression are common to quiescent and senescent cells (for a recent review see [5]) and quiescent neuronal stem cells, NSCs, in the subventricular zone have enlarged lysosome compared to activated NSCs [6]. Cell senescence is also characterized by the secretion of specific growth factors and cytokines, collectively defined as the senescence-associated secretory phenotype, SASP, which is induced as a delayed response to the pro-senescence stimuli [7,8]. Via autocrine and paracrine mechanisms, the SASP contributes to stabilize the senescent phenotype that normally becomes irreversible even after cessation of the senescence-inducing stimuli [9].

**Figure 1:**
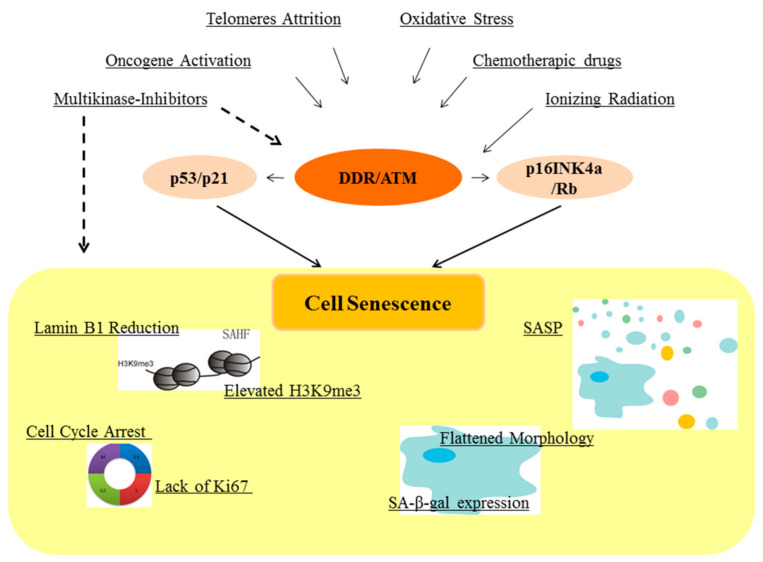
Induction of cell senescence. Cell-autonomous and non-cell-autonomous stimuli contribute to induce senescence through DNA damage response, DDR, epigenetic modifications (presence of senescence associated heterochromatin foci, SAHF) and ATM kinase activation. Senescent phenotype is characterized by flattened cell morphology, SA-β gal expression, cell cycle inhibition, epigenetic modifications, and by a peculiar secretome, the Senescence-Associated Secretory Phenotype (SASP).

The main cell-autonomous mechanisms that induce senescence of preneoplastic cells are telomeres attrition, which occurs upon clonal expansion in the absence of a functioning telomerase [10] and oncogene-induced senescence, OIS, originally described in primary cell cultures by Serrano et al. [11], and later confirmed to occur in vivo [12,13]. Beside overexpression or activating mutation of oncogenes, cell senescence may be caused by loss of tumor suppressors [14,15], through molecular pathways not always easily attributable to oncogene activation (see for instance [16]). Both telomere dysfunctions [17,18] and OIS [19] activate the cellular DNA damage response, DDR, which is necessary for senescence induction [17,19,20]. DDR, in turn leads to a temporary arrest of cell cycling followed by a persistent cell cycle arrest through p53/p21- and p16INK4a/Rb-regulated pathways. DDR appears to be necessary for senescence induction, but other pathways contribute to the acquisition of a mature senescent phenotype. For instance, a non-canonical DDR, which requires the ATM kinase but not its enzymatic activity, is required for the recruitment of NF-kB on the promoter of SASP genes and the delayed expression of a mature SASP [7]. About twenty years ago, chemotherapeutic drugs and X-rays irradiation used for cancer treatment were shown to induce cellular senescence in several human tumor cell lines [21] and in vivo [22,23]. Both treatments cause DNA damage, triggering cellular responses aimed at repairing the DNA via two major routes, non-homologous end joining (NHEJ) and homologous recombination (HR) [24]. Nevertheless, DNA damage may persist, resulting in halting transcription and replication, limiting cellular functionality, and eventually promoting cellular senescence or apoptosis. Since then, the research on therapy-induced senescence has acquired growing importance especially when also targeted therapies, like those inhibiting kinases, were shown to induce senescence [25]. As discussed in more detail in the next section, chemicals and antibodies inhibiting receptor tyrosine kinases for cancer therapy may trigger cell senescence (reviewed in [26]). The same holds true for inhibitors of serine/threonine kinase, which act downstream of growth factor receptors (e.g., the RAF/MEK/ERK cascade) or those like CDK4/6, which regulate cell cycle progression (reviewed in [27]). It is not clear if also these targeted therapies require activation of DDR for their pro-senescence action [28,29]. In general, not only DNA damage but also many kinds of cellular dysfunctions, like an imbalance of signaling, oxidative stress, proteotoxicity, altered chromatin structure result in cellular senescence, if unrepairable [1] (Figure 1). Treatments which cause DNA damage and cell stress induce senescence also in non-transformed cells mirroring the effects of aging. Non transformed senescent cells may have important consequences on cancer progression. When senescent stromal cells accumulate within the tumor mass, they alter the tumor microenvironment in different ways which may result in either pro or anti tumoral effects. In a recent paper [30], prostate stromal cells were shown to secrete an increased quantity of extracellular vesicles, EV, whose content was also qualitatively different from those released by growing prostate stromal cells. EV from senescent stromal cells increase the aggressiveness of adjacent cancer cells by favoring drug resistance. Additionally, modification of Extracellular Matrix (ECM) degradation by senescent fibroblasts was shown to alter the permeability of lymphatic vasculature in the way of changing the number and location of melanoma metastases [31]. In general, senescent stromal cells within the tumor mass act essentially via the SASP modulating the microenvironment or directly acting on cancer cells. In addition, tissue dysfunction due to senescence of normal cells may affect the function of cancer supporting structures such as the vascular network. These topics will be specifically addressed in Section 3 and Section 5. The issue of senescence of cells of the immune system goes beyond the aims of this review and the reader is directed to the many excellent recent reviews.

## 2. Therapy-Induced Senescence

Cancer cells are commonly regarded as immortal: their continuous cell dividing ability is supported by telomerase activation, an almost ubiquitous feature of transformed cells compensating for telomere attrition, or, in a minority of cases, by alternative mechanisms of telomere maintenance [32]. Telomere maintenance is a necessary requisite allowing continuous cell division, but it is not sufficient. Following a series of different stresses, such as genotoxic stress, mitochondrial dysfunction [33] and endoplasmic reticulum stress [34], cancer cells might stop dividing and enter senescence. The exposure to clinical doses of radiation or chemotherapeutic drugs is a leading cause of cancer cells senescence. It is also responsible for local and systemic toxicity of cancer therapy because of induction of senescence of normal cells in the tumor microenvironment and in various organs. Therapy-induced senescence, TIS, substantially contributes to the efficacy of therapy; however, a small percentage of senescent cancer cells, which maintain elevated level of cdc-2, is able to resume proliferation upon cessation of therapy [35,36,37]. These cells contribute to the relapse of tumor, which may occur months or even years after an apparent successful outcome of the therapy. A possible mechanism of senescence escape involves DNA duplication in the absence of mitosis, leading to polyploidy and cell division by budding (neosis) [38]. Importantly, the acquisition of a senescent phenotype may reprogram cancer cells to acquire a cancer stem cell phenotype increasing tumor aggressiveness [39]. These cells which concur to tumor relapse were authentically senescent, and not quiescent as discussed in [37]; thus, senolytic treatments, (discussed below see paragraph 6) may provide a valid help to conventional cancer therapies.

### 2.1. IR-Induced Senescence

Ionizing radiation (IR) is widely used in cancer therapy. Although a precise prediction of cell response to radiation is not possible, the main pathways activated in response to IR are DDR and antioxidant systems. IR kill cancer cells causing extensive DNA damage. Cancer cells, in turn, activate DDR. There is a direct relationship between the extent of IR-induced DNA damage and the IR dose used for therapy. Further, there is an intrinsic variability of treatment susceptibility as a peculiarity of different tumor types. DDR involves a series of actors, the most important being the kinase ATM and p53. DNA damage is sensed by a number of proteins that transmit the message of warning to different transducers and effectors of the DNA damage response machinery. These proteins coordinate checkpoint regulation and block cell cycle progression to allow sufficient time for DNA repair. If the damage is too severe, cells can be either eliminated by cell death or induced to an irreversible exit from cell cycle at the G1 or G2 phase [40], therefore preventing inappropriate cell cycle progression in the presence of unrepaired damage. Following double strands break (DSB), proteins acting as sensors of DNA damage signal the lesion to ATM and ATR. These kinases act as mediators phosphorylating downstream targets, which in turn work as effectors in DSB repair, like histone H2AX and p53, or in cell cycle arrest, like the checkpoint proteins CHK1 and CHK2. Of note ATM and ATR directly or indirectly phosphorylate a large number of substrates in the range of hundreds [41]. The role of many of them remains to be fully understood. ATM and ATR regulate many pathways that may be involved with senescence induction (reviewed in [42]). Recently a role of ATM promoting SASP has been described [43]. Authors demonstrated that ATM and downstream effectors are persistently activated in aged mice, together with NF-KB, a transcription factor important for driving senescence and aging. ATM inhibition resulted in reduced cellular senescence and downregulated NF-KB activation in cell culture, and reduced senescence and SASP marker expression in a mouse model of human progeroid syndrome.

The extent of DNA damage repair by downstream effectors regulates cell fate, triggering premature cell senescence or apoptosis [44]. Some evidence supports a role of p53 protein in determining cell fate upon IR exposure: cell senescence of breast cancer cells seems to require a functional p53 [45]. Recently, a role in cell fate choice, senescence or apoptosis, in IR-exposed cancer cells, was demonstrated for securin, the multifunctional protein involved in DNA replication and repair [46]: colon cancer cells with wild type securin undergo apoptosis upon IR exposure. Conversely, in securin-deprived cells, IR trigger senescence [47]. Glioblastoma cells choose their fate upon IR exposure, senescence or apoptosis, according to PTEN status: PTEN deficiency forces the equilibrium towards senescence, while PTEN proficiency leads to apoptosis [48]. In lung cancer cells, in turn, IR-induced senescence appears to be regulated by miR-34a [49].

As mentioned above, SASP composition can vary according to the senescence-causing stimulus. In vitro, cells exposed to IR showed to induce SASP after a long delay with respect to cells induced to senescence by different stimuli [50].

### 2.2. Chemotherapy-Induced Senescence

Chemotherapy is a well-known inducer of senescence too. When used at clinical concentrations, most genotoxic drugs trigger cellular senescence of cancer as well as of tumor microenvironment cells [21,51,52]. More than twenty years ago, a number of drugs targeting DNA replication and cell cycle progression were recognized as senescence inducers [21,53]. Further, doxorubicin was demonstrated to trigger senescence in vivo, as addressed analyzing tumor specimens from doxorubicin-treated cancer patients [22]. Drug-induced premature senescence is characterized by the peculiar hallmarks of cellular senescence, such as β-galactosidase expression and arrest of cell cycle. IR and DNA-damaging drugs trigger cellular senescence through the activation of DDR. As previously mentioned, DDR generally induces cell senescence via p53/p21- and p16INK4a/Rb-regulated pathways. However, p53 and Rb are often inactivated in advanced cancers, suggesting that, in the tug of war between oncogenes and tumor suppressors, alternative pathways to senescence may be activated by DNA damage [54]. For instance, the Tap63 isoform of the p53 homolog p63 was demonstrated to induce senescence in response to oncogene activation [55] and may contribute to TIS as well. Conversely, the cGAS/STING (cyclic GMP-AMP synthase and STimulator of INterferon Genes) pathway (see below Section 5) can induce senescence upon release of nuclear DNA fragments in the cytoplasm [56]. It is likely that others, still to be clarified, mechanisms of p53 and Rb-independent cell senescence exist.

### 2.3. Kinase Inhibitor-Induced Senescence

Not only DNA damage-inducing agents are able to cause premature cell senescence. Molecules affecting kinase activity can trigger cellular senescence as well. Kinases mediate a wide variety of physiological and pathological processes, like proliferation, differentiation, development, migration, apoptosis, inflammation which may affect tumorigenesis and tumor progression [57]. Up to 33% of drug discovery efforts focus on kinases, with about 175 orally effective protein kinase inhibitors in clinical trials worldwide (for a complete and updated list visit: www.icoa.fr/pkidb/). Currently, there are 52 FDA approved therapeutics, targeting 20 different kinases. Most kinase-targeting drugs inhibit tyrosine kinases, and relatively few inhibit serine/threonine kinases (B-RAF inhibitor: vemurafenib; MEK1/2 inhibitor: trametinib; cyclin dependent kinase inhibitor: palbociclib). Among kinase inhibitors, drugs targeting tyrosine kinase receptors have been showing clinical efficacy for almost twenty years. These inhibitors target the kinase active site preventing phosphorylation of their downstream effectors, typically involved in cell proliferation or angiogenesis [58]. A pro-senescence activity of kinase inhibitors has been widely described (for a recent review see [25]). Imatinib was the first kinase inhibitor to enter in the clinical management of tumor patients. It is approved for the treatment of chronic myeloid leukemia (CML) and its main target is chimeric kinase BCR-ABL. Imatinib-dependent suppression of tumor growth is exerted through both apoptosis and senescence [59]. Another classical signaling pathway targeted by kinase inhibitors for cancer treatment is the epidermal growth factor receptor (EGFR) pathway. There are a number of small molecule inhibitors of EGFR kinase activity (gefitinib, erlotinib, afatinib, osimertinib and lapanitib) and a senescence response to all of them has been reported in different model systems [60,61,62,63,64].

The Vascular Endothelial Growth Factor Receptors, VEGFRs, have tyrosine kinase activity and are, therefore, druggable by small kinase inhibitors. Several molecules have been described with a VEGFR inhibitory potential, including sunitinib, sorafenib, pazopanib, vandetanib, regorafenib, axitinib, lenvatinib and regorafenib. Most of them are multikinase inhibitors, since they target a panel of kinases, although with different specificities [29,65,66,67,68,69]. Most of these drugs induce senescence through activation of the DDR pathway. Interestingly, axitinib seems to induce cancer cell senescence following two possible routes: a chronic exposure induces DNA damage, in renal cell carcinoma and in glioblastoma cell lines, eventually leading to activation of double strand break repair pathways and senescence [66,69]. Conversely, transient exposure to axitinib induces cell senescence, characterized by an increased percentage of β-galactosidase positive cells, in glioblastoma cells in vitro, with increased Reactive Oxygen Species (ROS) and modification of SASP, but with no detectable activation of DDR [66]. Overall, the impact of targeting kinase receptors shows a certain variability, and we might speculate that the outcome might depend on a series of variables, ranging from cell type to exposure time and dose, up to off target effects. 

## 3. Therapy-Induced Senescence of Tumor Vasculature

Folkman, in his seminal article in 1971 [70], was the first to stress the importance of angiogenesis, the process of new blood vessels formation, in the progression of solid cancers. Since then, more than 60,000 papers have been published on this topic. Tumor angiogenesis is a complex phenomenon starting with the angiogenic switch, that is the imbalance between pro- and anti-angiogenic factors triggering tumor vascularization [71,72]. The process requires a series of steps: endothelial cells proliferation, extracellular matrix degradation and cellular migration toward the growing tumor mass. The master regulator of angiogenic phenomena is VEGF (Vascular Endothelial Growth Factor). Angiogenesis participates in several pathways, which shape the evolution of cancers: it is essential for delivering nutrients and oxygen to the tumor microenvironment and for removing waste products, it is important for cancer cell dissemination and metastasis formation [71]. Anti-angiogenic treatments have, as their main target, endothelial cells that are the principal component of vascular vessels and line the interior surface of cancer blood vessels. Many of these treatments target growth factors/growth factor receptors, which promote endothelial growth and maturation; above all, members of the VEGF/VEGFR family [73]. Importantly, VEGF acts not only on endothelial cells but also on those cancer cells that express VEGFR [74], thus potentially providing a double benefit of antiangiogenic therapies. Bevacizumab, a humanized monoclonal antibody targeting VEGF, was the first angiogenic inhibitor approved by the Food and Drug Administration [75] in 2004. It was initially approved for metastatic colorectal cancer in combination with standard chemotherapy. Its indications now include metastatic breast cancer, non-small-cell lung cancer, glioblastoma, renal cell carcinoma, ovarian cancer and cervical cancer (for an updated review, see [76]). Although Bevacizumab is a key agent in anti-angiogenic therapy, either alone or in combination with chemotherapy, several concerns were raised about its real benefits in the brain tumor glioblastoma. Bevacizumab administration extends the patients progression free survival but does not seem to significantly increase their overall survival [77]. In glioblastoma, Bevacizumab may trigger a phenotypic change of the tumor that undergoes a gliomatosis-like growth pattern. This infiltrative shift occurs mostly along perivascular spaces and relies on the over-expression of PLXDC1 (Plexin Domain Containing 1) by tumor cells and on the restoration of the endothelial component of blood brain barrier [78]. When targeting tumor angiogenesis, we should consider that a functional tumor vasculature allows the access in the tumor mass of therapeutic drugs and cells of the immune system. Additionally, hypoxia, which follows disruption of tumor vasculature, may select for more aggressive cancer subpopulations [79]. In addition to their structural role in neovascularization, endothelial cells are engaged in a constant dialog with tumor cells via secreted and surface anchored proteins and microvesicles. For instance, endothelial cells constitute a cancer stem cell niche in medulloblastomas and glioblastomas [80], possibly via signaling through NOTCH [81] and secretion of interleukin-8 (IL-8) [82]. 

Endothelial cells senescence upon exposure to radiation and the ensuing severe pathological consequence, has been extensively described (for a recent review see [83]) and the transcriptional profile of endothelial cells exposed to distinct senescence inducing agents has been studied [84,85]. On the other hand, tumor might circumvent the limits to neovascularization derived from a senescent microenvironment. In glioblastoma recurring following radiotherapy, brain endothelium undergoes radiation-induced senescence eventually resulting in the impairment of tumor neo-angiogenesis. This is not sufficient to limit tumor relapse since cancer cells may adopt the endothelial trans-differentiation as a supportive mechanism to build up its vasculature and bypass senescence-dependent reduction of tumor vessels formation [86].

We recently demonstrated that the VEGFRs inhibitor axitinib induces endothelial cell senescence through ROS accumulation and ATM activation, in the absence of DNA damage [29]. Although we also observed senescence induction and ROS increase by glioblastoma tumor cells exposed to axitinib, there is a substantial difference in the response of endothelial versus tumor cells. While endothelial cells senescence is prevented by concomitant administration of antioxidants or by ATM inhibition, the same does not hold true in cancer cells, whose commitment to senescence upon axitinib treatment is irreversible. This observation has the intrinsic consequence that antioxidants or ATM inhibitors might be taken into consideration to protect endothelial cells from axitinib-induced senescence. This would bring a double benefit: lowering of therapy adverse effects on normal cells, and tumor vessel normalization, a requisite allowing a more effective delivery of chemotherapy to the tumor mass. These studies need to be verified in vivo since we recently found that axitinib-induced senescence of endothelial cells may be modified by factors released by glioblastoma cells [87].

Therapy-induced senescence of endothelial cells has a double effect on cancer progression: from one side, it disturbs the vascular architecture and inhibits angiogenesis; from the other side, it perturbs the reciprocal influence between endothelial and cancer cells. The issue of SASP-mediated crosstalk between tumor cells and the surrounding microenvironment has a profound impact in determining tumor response to therapy. In a recent paper, for instance, Ruscetti et al. [88] describe an interesting dynamic interplay between cancer cells, drug-induced cell senescence, SASP, angiogenesis, immune cytotoxic CD8+T cells and checkpoint inhibitors. Briefly, inhibitors of MEK and KRAS kinases induce senescence of pancreatic ductal carcinoma cells; the associated SASP promotes angiogenesis and increases vascular permeability, which allows delivery of cytotoxic drugs to the tumor. Increased vascular permeability also promotes the influx into the tumor mass of CD8+T cells that, upon the administration of anti PD-1 checkpoint inhibitors, give a strong antitumor response. Conversely, Hwang et al. demonstrated an increased aggressiveness of breast cancer cells mediated by CXCL11 released as a SASP component of senescent endothelial cells [89].

## 4. Senescence: The Light Side or The Dark Side of the Force?

Through a permanent arrest of cell cycle division, senescence exerts a strong cell-autonomous tumor suppressor action. Senescence would be a tumor suppressor mechanism, alternative to apoptosis, to prevent growth of a defective cell population potentially detrimental for the organism. Although cell senescence contributes to the therapeutic properties of anticancer treatments, it is not without severe side effects because of local or systemic toxicity due to organ dysfunction and inflammation. In this respect, treatments that protect normal cells from senescence without altering the response of transformed cells [29] may widen the therapeutic windows of cancer therapies. However, acquisition of a full senescent phenotype is a long-lasting, multistage process, which may be reverted by loss of tumor suppressors [90,91]. Additionally, there are processes such as loss of epigenetic silencing of proliferation-promoting genes, which allow cells to escape even from terminal senescence [92] (for a review on the dynamic nature of senescence see [93]). As previously mentioned, cells that escape senescence may acquire a more stem-like phenotype and, thus, increase the population of cancer initiating cells, eventually contributing to a greater aggressiveness of the tumor [39]. It is also possible that senescent cells within the tumor are enriched for cancer stem cells. This make sense considering that senescence is a tumor suppressor mechanism which may preferentially target those cells more prone to cause tumor expansion and recurrence. As mentioned above, tissue damage and senescence promote cellular reprogramming in vivo [94].

## 5. SASP: An Anti- and Pro-Tumorigenic System

A main functional difference between senescence and apoptosis is that apoptotic cells are disposed without affecting neighbor cells or causing inflammation while senescent cells persist, have a vigorous metabolism [95,96], which sometimes sustain at expenses of neighbor cells, and act locally, and sometimes systemically, via their SASP. SASP composition changes with time, a process regulated by NOTCH1, assuming either an immunosuppressive or inflammatory function [97,98]. In addition, hypoxia affects the expression of SASP genes [84].

SASP may play both tumor suppressor and tumor promoter activities (reviewed in [99]). Importantly, the balance between these suppressor and promoter activities may vary with time during cancer evolution in response to the changing tumor microenvironment, to host innate and adaptive immunity as well as to therapeutic pressure. A transient SASP favors removal of transformed cells. If this event, even partially, fails because of an immunosuppressive tumor microenvironment, a persistent SASP may promote growth of residual cancer cells analogously to what happens for inflammation [100]. The different outcomes of SASP on tumor progression depend on the type of cancer [101] and on the composition of SASP, which, in turn, depends on the kind of senescence-causing stress. Pro-senescence stimuli activate SASP via different signaling pathways, which generally converge on the activation of NF-kB and C/EBPβ and may be fine-tuned by other transcription regulators (for a recent review see [98]).

Campisi and collaborators were the firsts to demonstrate that SASP differs according to the senescence inducing stimuli [33]. They showed that mitochondria dysfunctions cause a specific senescence phenotype, named mitochondrial dysfunction-associated senescence (MiDAS). MiDAS displayed typical markers of senescence such as SA-β-gal, decreased DNA synthesis, reduction of lamin B1, decreased nuclear localization of high mobility group protein B1 and elevated level of p21 protein. However, different from IR induced cell senescence, MiDAS failed to induce production of mRNAs for IL1β, CXCL1, CXCL2, IL-6, IL-8 and VEGF and MiDAS associated SASP lacked increased secretion of IL-1β, CXCL1, CXCL2, IL-6, IL-8, and VEGF. Importantly MiDAS associated SASP was shown to be present in vivo in a progeroid mouse model which accumulates mitochondrial dysfunction due to the expression of a mitochondrial DNA polymerase with reduced proofreading capability. They showed that several mitochondria stresses result in a SASP with reduced levels of a set of inflammatory cytokines, like IL1α, IL1β, IL6, and IL8, but not others, like CCL27 and TNF-α. Mechanistically, mitochondria dysfunctions lead to reduced NAD+/NADH ratio, causing activation of AMPK kinase, p53 phosphorylation on serine 15 and consequent arrest of cell cycle and cellular senescence. At the same time, p53 prevents the activation of NF-kB. The functional consequence of this finding needs yet to be fully understood. Recently, the cGAS-STING pathway was shown to be an important regulator of SASP. cGAS-STING pathway was originally characterized as a component of the innate immune system which recognizes pathogen associated intracellular (cytoplasmic) DNA (reviewed in [102]) and leads to the production of type I interferons and pro-inflammatory cytokines. Briefly, cytoplasmic DNA binds and activate a normally inactive cGAS. This leads to the synthesis of cyclic GMP-AMP (2’3’-GAMP) which is detected by STING, a transmembrane protein residing at the endoplasmic reticulum. Upon 2’3’-GAMP binding STING translocate to the Golgi and promote the autophosphorylation and activation of the TANK-binding kinase 1 TBK1 kinase, which causes phosphorylation and activation of the transcription factor interferon regulatory factor 3 and synthesis of interferons. It was recently shown that several prosenescent stimuli like oncogene activation, telomeric dysfunctions and DNA damage, cause the accumulation in the cytoplasm of nuclear DNA fragments that are recognized by the cGAS, leading to the expression of an inflammatory SASP [103,104,105]. Besides the classical signaling, which results in production of interferons, STING also activates the transcription factor NF-kB, which induces SASP [106]. Finally, a portion of cGAS was found to reside in the cell nucleus where it inhibits repair of double strand DNA brakes via homologous recombination [107,108]. For this inhibitory effect, the enzymatic activity of cGAS is dispensable. Presently, it is not known if and how this function of nuclear cGAS may affect DDR-dependent cell senescence.

**Figure 2:**
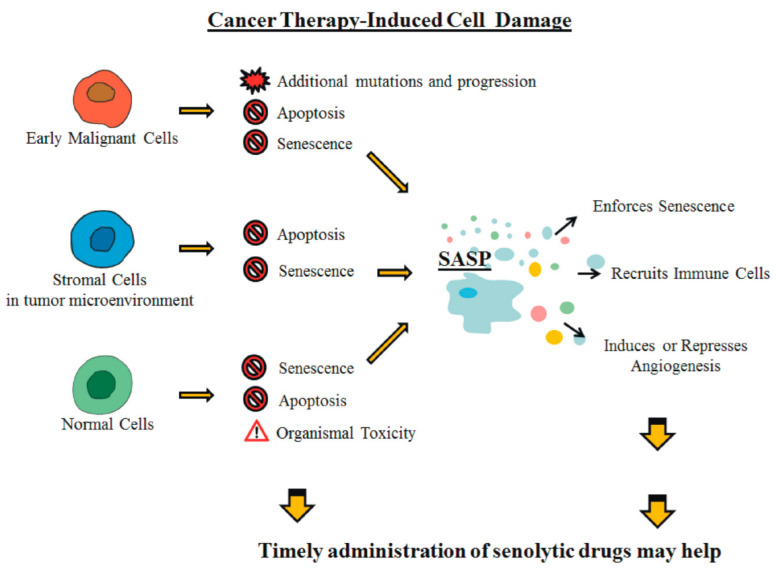
Cancer therapy-induced cell damage. IR, chemotherapy, as well as multikinase inhibitor-based therapies, induce widespread cell damage affecting proliferating tumor cells, stromal cells in the tumor microenvironment and normal cells in healthy tissues. This induces apoptosis or senescence and its associated SASP, that in turn amplifies senescence, recruits immune cells and modulates angiogenesis. Senolytic drugs may impact on cells (tumor or normal) response to SASP.

Tumor repressor functions of SASP include reinforcement of tumor cell senescence [8,109] and favoring clearance of premalignant cells via recruitment of immune cells, as initially demonstrated in [110,111]. Tumor promoting function of SASP include stimulation of cancer cell growth, initially observed in [111], promotion of epithelial to mesenchymal transition and increased cell migration and metastasis [50], drug resistance [112,113] and stemness [114]. Recently, senescent cells persisting after cancer therapy were suggested to promote carcinogenesis [115], which may explain the increased risk of tumor formation long time after radio- or chemotherapy (Figure 2).

## 6. Treatments that Alter Senescence

For all the reasons discussed above, many efforts are currently spent in attempts to maintain and promote the therapeutic effects of cell senescence and to curb and avoid its pro-tumoral activity. Accumulation of senescent cells leads to organ dysfunction and organ failure, thus representing a major side effect of anticancer therapies. In addition to its dual role in cancers, cell senescence contributes to organismal aging, and this is the reason why drugs that eliminate senescent cells, named senolytic drugs, have a huge therapeutic potential. A transgenic mouse where senescent cells can be selectively eliminated has been instrumental to demonstrate the potential beneficial effect of senolytic drugs. In brief, the expression of a recombinant caspase 8, which can be activated in response to the non-toxic drug AP20187, was put under the control of the promoter region of the INK4a gene, which is active in most senescent cells, being silent in non-senescent ones. Upon treatment with AP20187, senescent cells underwent apoptosis with a substantial reduction of precocious [116] and physiological [117] aging. Further, a transgenic mouse model, which allows disposal of senescent cells by a similar approach [118], has recently been used to demonstrate that therapy-induced senescence promotes a long lasting local and systemic inflammation and contributes to therapy toxicity [119]. Development of safe senolytic drugs requires the identification and targeting of pathways specific of senescent cells and essential for their survival (for a discussion on this topic see [120]). In this regard, it was recently demonstrated that urokinase-like plasminogen activator receptor, uPAR, represents a promising candidate for a senescent cell-specific marker expressed on the cell surface, which may be targeted via CAR-T therapy [121]. A detailed discussion on senolytic drugs goes beyond the scope of this review, and we direct the readers to the many excellent ones published on this topic [122,123]. In view of the previously mentioned dynamic nature of cell senescence and the evolving composition of SASP, which may shift from immunosuppressive to immune promoting, we envisage that the schedule of senolytic therapy must be carefully evaluated. It should be also considered that elimination of senescent cells may have serious adverse effects [124]. Rejuvenation is a possible second strategy to reverse the negative effects of cell senescence. In a very recent publication [125], it was shown that ectopic expression of three out of the four Yamanaka transcription factors (i.e., OCT4, SOX2 and KLF4; OSK) was sufficient to restore the epigenetic landscape which characterized young cells and that is lost in response of extensive damages and aging. This occurred without loss of cell identity or induction of teratomas or tissue dysplasia. No increase in incidence of tumor formation was observed. Rejuvenation of mouse retinal ganglionic cells via OSK expression promoted axon regeneration after injury and ameliorated vision in aged mice.

## 7. Conclusions

Senescence is an antagonistic pleiotropic genetic program (for a discussion on this topic see [126]), which contributes to tissue remodeling and to wound healing during early life, but promotes organismal dysfunctions which occur at later age due to accumulation of unrepaired cellular damage. OIS is essentially a tumor suppressor mechanism which prevents, sometimes for the entire organismal life, expansion of preneoplastic cells [12,13]. It is a cell-autonomous mechanism which, however, requires enforcement of cell cycle arrest by autocrine signaling provided by SASP factors [8,109,127]. More complex is the case for therapy-induced cell senescence, an unexpected response to treatments intended to kill cancerous cells. As mentioned before, genotoxic agents and targeted therapies generally induce senescence at concentrations below those required for inducing cell death. Since distribution and half-life of drugs in different organs vary, cell senescence is an almost unavoidable effect also of cytotoxic drugs. The greatest difficulty in predicting the efficacy and safety of a senescence-inducing therapy is due to the long-time persistence of senescent cells, being tumor cells or out-of-target normal cells. Although senescence prevents expansion of transformed cells, SASP with time may select for more aggressive phenotypes in tumors, especially those that are rapidly evolving and acquire mutations that allow escape from senescence-induced cell cycle arrest. A further element of complexity is due to the influence that SASP of senescent cancer and stromal cells have on the innate and adaptive immune response. Finally, cell senescence of endothelial cells affects angiogenesis, which promotes cancer growth and dissemination, but also delivery of therapeutic drugs and recruitment of immune cells. During evolution, senescence was probably selected because of its role in tissue remodeling and wound healing as a temporary-restricted mechanism that terminates with recruitment, via SASP, of immune cells and clearance of senescent cells [118,128]. During anticancer therapy, however, persisting populations of senescent cells may promote a perduring inflammation with organismal toxicity and potential tumor promoting effects. Ideally, senescence-inducing therapies should be complemented with senolytic drugs given at the appropriate time to maximize the anticancer effects of cell senescence and minimize its tumor promoting effects and systemic toxicity. Such an option, whose implementation is extremely challenging, is currently intensively investigated. Other approaches like cell rejuvenation are now at their infancy and it is premature to discuss their possible use in combination with senescence-inducing therapies.
